# Deciphering Angiogenic Drivers in Hepatocellular Carcinoma: From Prognostic Signature Construction to Genistein‐Mediated Inhibition

**DOI:** 10.1111/jcmm.71203

**Published:** 2026-05-24

**Authors:** Hao Zheng, Shengwei Tang, Ruida Shi, Zichuan Yu, Ruiyu Zhang, Xiaoyu Qu, Hao Wan, Chenshuo Fang, Xin Wang, Ye Zhu, Da Huang, Gen Chen

**Affiliations:** ^1^ Department of Hepatobiliary Surgery III Guizhou Provincial People's Hospital Guiyang China; ^2^ Second College of Clinical Medicine Nanchang University Nanchang China; ^3^ Department of Thyroid Surgery Second Affiliated Hospital of Nanchang University Nanchang Jiangxi Province China

**Keywords:** angiogenesis, biomarker, chemokine, hepatocellular carcinoma, pharmacology

## Abstract

Hepatocellular carcinoma (HCC) is an aggressive malignancy with poor prognosis and a lack of reliable biomarkers. In this study, we integrated weighted gene co‐expression network analysis, machine‐learning‐based prognosticmodelling, single‐cell RNA sequencing, spatial transcriptomics, and molecular docking to identify angiogenesis‐related drivers in HCC. SCAF1 was identified as a hub gene and was significantly upregulated in HCC tissues compared with normal liver tissues. High SCAF1 expression was closely associated with advanced clinicopathological features and unfavourable survival outcomes. The prognostic model constructed by machine learning showed robust predictive performance in both training and validation cohorts, and decision curve analysis supported its clinical utility. Single‐cell and spatial transcriptomic analyses further suggested that SCAF1 was mainly associated with endothelial and immune‐related features of the tumour microenvironment. Functional assays showed that SCAF1 knockdown suppressed HCC cell migration, invasion, VEGFA expression, and angiogenic capacity in vitro. Mechanistically, SCAF1 was negatively correlated with anti‐angiogenic chemokines, including CCL19, CCL14, and CCL11, indicating a role in shaping a pro‐angiogenic microenvironment. In addition, molecular docking identified genistein as a promising compound with stable binding to SCAF1. Experimental validation showed that genistein reduced SCAF1 and VEGFA expression and inhibited HCC cell proliferation, migration, invasion, and tube formation. Collectively, these findings suggest that SCAF1 is a novel prognostic and angiogenesis‐related biomarker in HCC and a potential therapeutic target for genistein‐based intervention.

## 1 Introduction

Hepatocellular carcinoma (HCC) is a solid tumour with a poor prognosis and fast development due to its high vascularisation [1, 2]. Its five‐year survival rate is merely 20% [3]. Based on existing studies, vascular endothelial growth factor (VEGF) is a signalling pathway capable of triggering downstream cellular pathways through phosphorylation cascades, ultimately leading to endothelial proliferation and migration [4, 5, 6]. It promotes tumour angiogenesis and development by stimulating endothelial cell proliferation and migration, closely associated with tumour angiogenesis, rapid growth, and dissemination [7, 8, 9]. The VEGF signalling pathway is collectively regulated by various angiogenesis‐related factors. Among these VEGFs, VEGFA is produced by tumour‐associated macrophages (TAMs) and manipulates tumour cell invasion and migration by enhancing VEGFA signalling transduction and bioavailability [10, 11].

An increasing body of research indicates that numerous chemokines can modulate tumour angiogenesis levels [12, 13]. Chemokines constitute a superfamily composed of small proteins capable of specifically binding to downstream cell G‐protein‐coupled receptors, altering cell secretion activity, and thereby regulating various cellular functions. As a result, chemokines are widely involved in the development, metastasis, and angiogenesis of tumours [14, 15, 16]. Chemokine CC ligand 19 (CCL19), also known as macrophage inflammatory protein 3‐beta (MIP‐3b), has been shown in recent studies to suppress the expression level of VEGFA in colorectal cancer, thereby inhibiting tumour occurrence, metastasis, and angiogenesis [17]. It is not evident, nevertheless, whether CCL19 controls tumour angiogenesis in HCC.

Human SR‐related CTD‐associated factor 1 (SCAF1), also known as SR‐A1, is a new member of the human SR (Ser/Arg‐rich) pre‐mRNA splicing factor superfamily [18]. It participates in the pre‐mRNA splicing process, thereby affecting the level of interferon regulatory factor 3 (IRF3) in serum, subsequently influencing the incidence and progression of cancer. Evidence suggests that elevated SCAF1 expression significantly increases the grading and staging of ovarian tumours and lowers ovarian cancer patients' overall survival (OS) and progression‐free survival (PFS), leading to a poorer prognosis [19]. Furthermore, research has indicated that elevated levels of SCAF1 can lead to decreased disease‐free survival (DFS) in colorectal cancer patients, leading to a dismal outlook for those with colorectal cancer [20]. However, there are no reports on the expression and related regulatory mechanisms of SCAF1 in HCC.

In this study, we identified the hub gene SCAF1. By using western blot and quantitative reverse transcription (qRT)‐PCR, we discovered that SCAF1 was elevated in HCC relative to normal liver tissue. Furthermore, we discovered a correlation between the clinical pathological characteristics and patient survival and the expression level of SCAF1. Furthermore, by encouraging angiogenesis, our research showed that SCAF1 might improve the invasive and migrating capacities of HCCLM3 cells. Chemokine experiments showed that SCAF1 could downregulate the expression of chemokines such as CCL19 and promote angiogenesis. Meanwhile, we also screened out the potential small molecule genistein that can block VEGF. According to our research, genistein can stop HCCLM3 cells from proliferating and lower VEGFA expression levels by reducing SCAF1 expression. This prevents HCCLM3 cells from being invasive and migrating. In summary, our research has identified a new biomarker associated with angiogenesis and invasion in HCC, which can be used for the diagnosis of HCC patients. Additionally, we have identified genistein, a small molecule that can effectively inhibit HCC angiogenesis.

## 2 Materials and Methods

### 2.1 Data Acquisition and Processing

The TCGA and ICGC databases provided the clinical samples and mRNA expression data for Hepatocellular carcinoma (HCC). In this work, 50 normal samples and 374 HCC samples were chosen for gene expression profile analysis from the Cancer Genome Atlas (TCGA) database, while 202 normal samples and 243 HCC samples were chosen from the International Cancer Genome Consortium (ICGC) database. Additionally, clinical data from 377 HCC patients were collected from TCGA for clinical‐pathological feature analysis.

### 2.2 Weighted Gene Co‐Expression Network Analysis (WGCNA)

WGCNA is an R package that identifies a group of genes exhibiting synchronous changes [21]. Initially, the optimal soft‐thresholding power (β) was set to 18 to follow a scale‐free network distribution. Gene modules were determined using hierarchical clustering. Subsequently, key modules were selected based on the correlation analysis of clinical samples. The genetic connectivity, measuring the degree of correlation between one gene and another, was calculated. Core genes were those with high connectivity within the main module.

### 2.3 Signature Generated From Machine Learning‐Based Integrative Approaches

To develop a liver cancer‐related prognostic model, we integrated 10 machine learning algorithms and 101 algorithm combinations. The integrative algorithms included random survival forest (RSF), elastic network (Enet), Lasso, Ridge, stepwise Cox, CoxBoost, partial least squares regression for Cox (plsRcox), supervised principal components (SuperPC), generalised boosted regression modelling (GBM), and survival support vector machine (survival‐SVM) [22]. The signature generation procedure was as follows: (a) Univariate Cox regression identified prognostic genes in the TCGA cohort; (b) Then, 101 algorithm combinations were performed on the prognostic genes to fit prediction models based on the leave‐one‐out cross‐validation (LOOCV) framework in the TCGA cohort; (c) All models were detected in two validation datasets (ICGC, GSE43619); (d) For each model, the Harrell's concordance index (C‐index) was calculated across all validation datasets, and the model with the highest average C‐index was considered optimal.

### 2.4 Dimensionality Reduction and Clustering

We downloaded the information of 34 HCC patients from GSE189903 and used the Seurat R tool to handle the scRNA‐seq dataset [23]. Cells were filtered (genes/cell 300–7,000; UMI > 1,000 with the top 3% highest‐UMI cells removed; mt_percent < 10%; HB_percent < 3%). Filtered data were normalised, clustered, and visualised by UMAP; FindClusters identified 19 clusters, annotated via SingleR. Cluster markers were detected with FindAllMarkers (logFC > 1, adj. *p* < 0.05).

### 2.5 TIMER Database Analysis

TIMER (https://cistrome.shinyapps.io/timer/), an integrated database, is used to analyse tumour immune infiltration [24]. The associations between immune cell infiltration, SCAF1 mRNA expression, and copy number changes in HCC samples were predicted using the “Gene” and “SCNA” modules. Gene co‐expression was displayed via the “Correlation” module. We also looked at the connection between different immune cell infiltrations in pan‐cancer tissues and SCAF1.

### 2.6 Gene Set Enrichment Analysis (GSEA)

GSEA is a gene set enrichment analysis method based on a set of genes [25]. RNA sequencing data related to HCC were obtained from TCGA. Using the following fundamental threshold values, we ranked the samples and obtained normalised enrichment scores (NES) by splitting them into two groups based on the expression of SCAF1 using the GSEA program (version 4.0.3): 0.05 FDR q‐value and 0.05 *p* value.

### 2.7 TCMSP

TCMSP (https://old.tcmsp‐e.com/tcmsp.php, Traditional Chinese Medicine Systems Pharmacology) is built upon the framework of traditional Chinese medicine pharmacology [26]. It contains 499 Chinese medications with 29,384 chemicals, 3311 targets, and 837 associated disorders that are listed in the Chinese Pharmacopoeia. Based on this, we searched for herbal ingredients capable of treating HCC by inhibiting angiogenesis: Genistein, rutaecarpine, bifendate, hirsutasideB, and genistein.

### 2.8 PubChem

PubChem (https://pubchem.ncbi.nlm.nih.gov/) gathers chemical information from hundreds of data integration sources (as of 6 September 2022, 872 sources). Most of the chemicals contained in PubChem are small molecules, and their chemical structures are provided. We obtained the chemical structures of these 5 herbal ingredients from PubChem for further analysis.

### 2.9 Protein Structure and Docking Analysis

The RCSB Protein Data Bank (https://mcule.com/dashboard/) collected three‐dimensional structural information about proteins. We obtained the molecular structure of the SCAF1 protein from this database. Using the 1‐Click Docking web server, we investigated the binding modes of SCAF1 with the molecules of 5 traditional Chinese medicine ingredients and used PYMOL (2.5.5) tovisualise, analyse, and map them.

### 2.10 TISIDB

TISIDB (http://cis.hku.hk/TISIDB/) is a tumour immune analysis database that integrates data from five major types of tumour immune‐related data [27]. Based on this, we investigated the association of SCAF1 with six immune system components (lymphocytes, immune modulators, convergent factors, etc.) in HCC.

### 2.11 Statistical Analysis

The R program (version 4.3.0) was used for statistical analysis. Differential expression of SCAF1 was detected by a rank‐sum test using the “limma” and “beeswarm” packages. The “survival” and “survminer” packages were used to conduct multivariate Cox regression analysis to identify significant predictors (*p* < 0.05). ROC curves were created using the SurvivalROC package (version 3.3.1) to assess the ability of SCAF1 expression levels to predict 1, 3, and 5‐year survival rates. Survival scenarios for SCAF1 were created in prevalent cancers using the “Survival” and “SorvMiner” packages. The tumour mutation burden (TMB) of each tumour was calculated using the TMB function in the MAfTools package. The “pheatmap” package was used to investigate the relationship between SCAF1 expression and immune checkpoint genes. Unique states of tumour mutation burden were revealed by combining sample TMB and gene expression data.

### 2.12 Cell Culture

Huh7, HCCLM3, and SMCC7721 HCC cell lines were acquired from the National Collection of Authenticated Cell Cultures. Except for HCCLM3 cells, which were grown in RPMI‐1640 media, all cell lines were grown in DMEM enhanced with 10% FBS and 1% penicillin–streptomycin. Every cell was grown in an incubator with 5% CO2 and a humidifier set at 37°C.

### 2.13 Western Blot Analysis and Antibodies

Prepare the total protein extracts according to the method described previously. Using RIPA buffer (Beyotime, Shanghai, China) to extract protein, a buffer mixture containing protease and inhibitors (Thermo Fisher Scientific, New York, USA). The monoclonal antibodies used in the experiment were: Anti‐SCAF1 (1:1000, Solarbio, K112765P); anti‐VEGFA (1:1000, Abcam, ab46154); and anti‐Tubulin (1:1000, Proteintech, 11,224–1‐AP).

### 2.14 Quantitative Real‐Time PCR (qRT‐PCR)

To assess the relative RNA levels of genes, quantitative real‐time PCR was used. Total RNA was extracted using the standard Trizol‐based protocol (Invitrogen, USA). Reverse transcription of RNA was performed using the PrimeScript RT Reagent Kit (Invitrogen, USA), and qPCR was conducted using SYBR Premix Ex Taq (TaKaRa, China), in accordance with the manufacturer's instructions. Table S1 contained details on the gene‐specific primers.

### 2.15 Transwell Migration Assay

Stable transfected cells were utilised for tests of invasion and migration after 48 h of seeding. Serum‐free media was used to plate 6 × 10^4^ cells in the upper chamber for migration, while 1 × 10^5^ cells were plated in a Matrigel‐coated chamber (BD Biosciences) in preparation for invasion. To get rid of unmigrated cells, the top surface of the membrane was gently wiped 24 h (for migration) or 48 h (for invasion) after seeding. Fixing and staining the migrating cells with 0.1% crystal violet was done. Five randomly chosen microscopic fields were used to count the cells using a light microscope equipped with a DP70 CCD system (Olympus Corp, Japan).

### 2.16 Tube Formation Assay

Incubate HCCLM3 cells on six‐well slides for 24 h. The medium from the cells was collected and used as the medium for the tube formation assay. Pipette tips and 96‐well plates were pre‐cooled. Apply 50 μL of Matrigel to the wells of the 96‐well plate and incubate for 30 min. Inoculate the suspension of treated Human Umbilical Vein Endothelial Cells (HUVECs) into Matrigel‐lined 96‐well plates (5 × 10^4^ cells/well). Following an 8‐h incubation period at 37°C, use the microscope to evaluate angiogenesis. Branching density and tube length were measured by randomly choosing three locations per well.

### 2.17 CCK8 Assay

Transfected cells were plated into each well of 96‐well plates after being cultured for 48 h to assess CCK8. In total, 10 μL of CCK8 reagent was added to each well and incubated for 2 h at 37°C after 24, 48, 72, 96, and 120 h. With the use of a microplate reader (ELX‐800; Bio‐Tek, Winooski, VT, USA), the absorbance value was determined at 450 nm.

## 3 Result

### 3.1 Establishment of Prognostic Models in HCC

**FIGURE 1:**
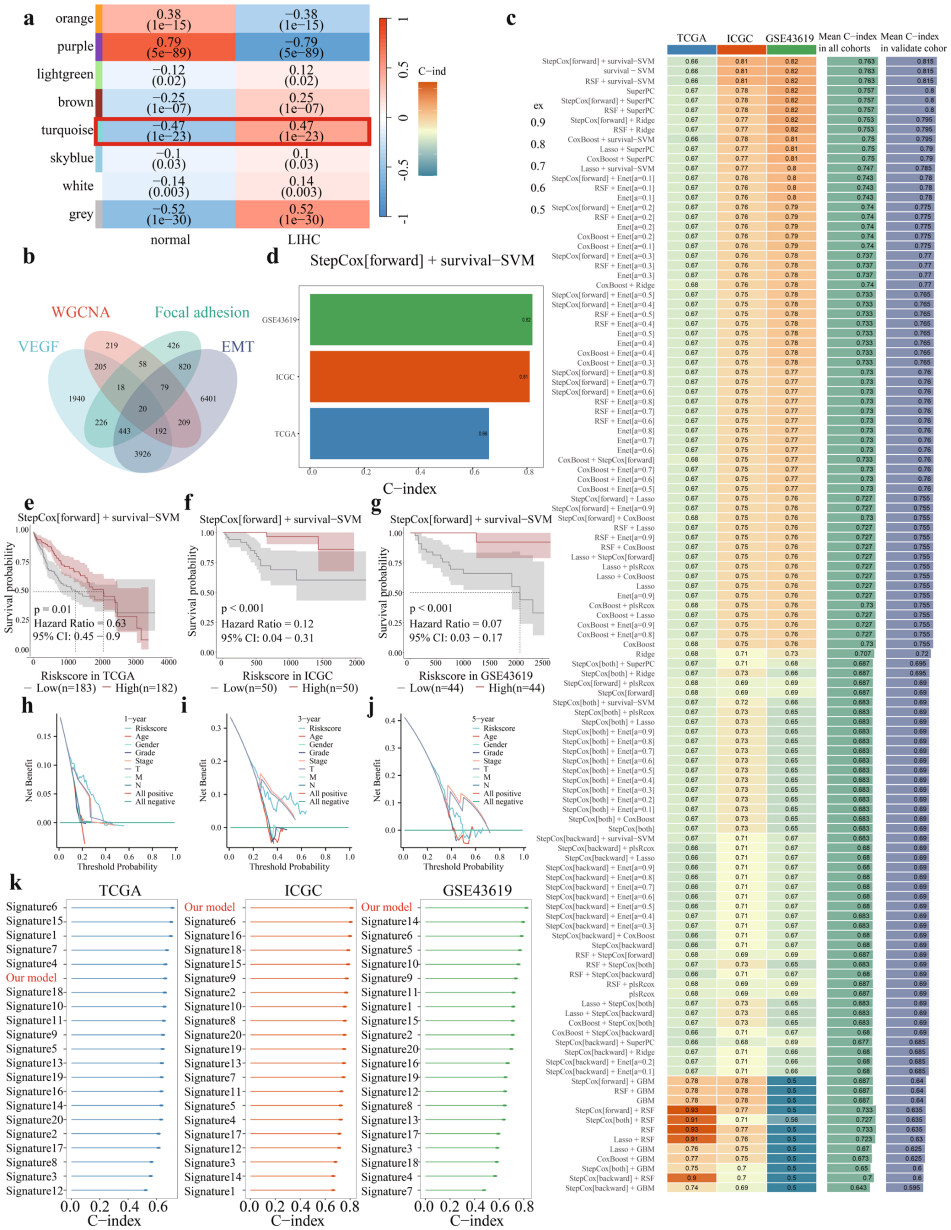
Establishment of prognostic models in HCC. (a) Heatmap of correlations between genetic modules and clinical features. (b) WGCNA was used in conjunction with genes linked to VEGF, focal adhesion, and EMT to screen out 20 genes, as seen in the Venn diagram. (c) The LOOCV framework yielded 101 prediction models in total, and each model's C‐index across all validation datasets was then computed. (d) The Stepcox[forward] optimal model's average C‐index in conjunction with survival‐SVM over the three datasets. Stepcox[forward] integrated with survival‐SVMs. (e–g) DCA curves for the three datasets, TCGA (e), ICGC (f), and GSE43619 (g). (h–j) Kaplan–Meier curves for the three datasets, TCGA (h), ICGC (i), and GSE43619 (j). (k) Comparison of C‐index values of different models.

To confirm the key genes in HCC, we confirmed a series of genes that were upregulated in HCC by WGCNA. After that, β = 18 was selected as the soft threshold for implementing a scale‐free network (Figure S1a). Using the dynamic tree cutting package, 29 modules were defined (Figure S1b). To identify gene modules associated with HCC, we constructed a HCC‐related heatmap, where the turquoise module exhibited the strongest correlation with HCC (Figure 1a). The gene distribution results in the turquoise module displayed that HCC and Module membership (MM) were highly associated, suggesting that genes in this module were highly significantly correlated with HCC (Figure S1c). Subsequently, we intersected VEGF‐related genes, focal adhesion‐related genes, and EMT‐related genes with genes in the turquoise module, resulting in 20 genes identified through a VN map (Figure 1b). Using the LOOCV framework, we fitted 101 predictive models to the TCGA dataset. We then computed each model's C‐index across all validation datasets (Figure 1c). With the highest average C‐index (0.763) and a much higher average C‐index in the validation sets, it is evident that the best model combines Stepcox[forward] with survival‐SVM (Figure 1d). Additionally, to evaluate the clinical net benefit of the model across different time points, decision curve analysis (DCA) was performed for 1‐, 3‐, and 5‐year survival (Figure 1e–g). Patients with higher risk scores were classified as the high‐risk group, whereas those with lower risk scores were classified as the low‐risk group, representing the upper and lower halves of the risk‐score distribution, respectively. Next, based on the ideal cut‐off value identified by the Survminer software, all patients were divided into high‐ and low‐risk groups. In the TCGA training dataset and two validation datasets, patients in the high‐risk group had significantly worse overall survival (OS) than those in the low‐risk group, as shown in (Figure 1h–j) (all *p* < 0.05). In addition, we compared the C‐index of our model with several previously reported models and found that the C‐index of our model ranked high across all three datasets (Figure 1k).

### 3.2 Single‐Cell Analysis of HCC

**FIGURE 2:**
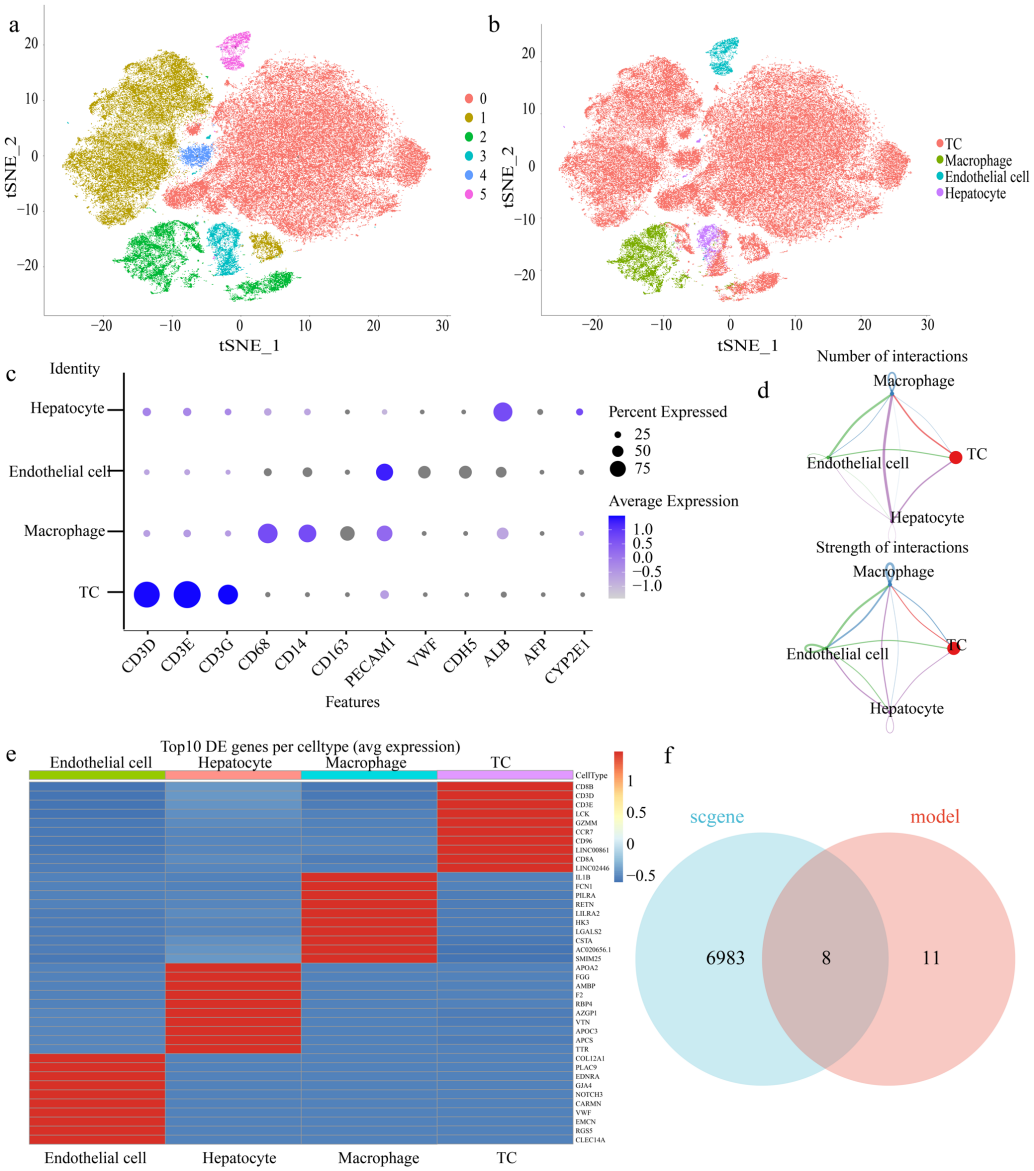
Single‐cell analysis of HCC: (a, b) Using single‐cell RNA sequencing to investigate the tumour microenvironment of hepatocellular carcinoma (HCC). A t‐SNE‐projected visualisation of sample clustering results. Cell clustering results within the samples are visualised. (c) Representative gene expression levels in each cell are visualised. (d) Cell interactions arevisualised. (e) The top 10 genes that differ between cells are displayed. (f) Visualisation shows the interaction between machine learning genes and differential genes between cells.

We downloaded information from the GEO database and created single‐cell gene expression profiles to investigate the tumour microenvironment features of hepatocellular carcinoma (HCC). Following quality control, as illustrated in (Figure 2a,b) and (Figure S3a), we identified and annotated six cell clusters and four cell types: T cells (TC), macrophages, endothelial cells, and hepatocytes. The volcano plot reveals the highly differentially expressed genes across distinct cell populations (Figure S3b). A bubble plot is used in Figure 2c to show the percentage and normalised expression levels of particular genes. We next used cell interaction analysis to visualise the interaction strengths between the four cell kinds (Figure 2d). Additionally, we examined these cells' highly variable genes and produced a heatmap showing the variations in genes between the various cell types (Figure 2e). Ultimately, we identified eight target genes by intersecting the cell‐specific differential genes with genes from a machine learning model. The results were displayed using a Venn diagram (Figure 2f). Among them, liver cancer has not yet been linked to SCAF1.

### 3.3 Clinical Predictive Significance of SCAF1

**FIGURE 3:**
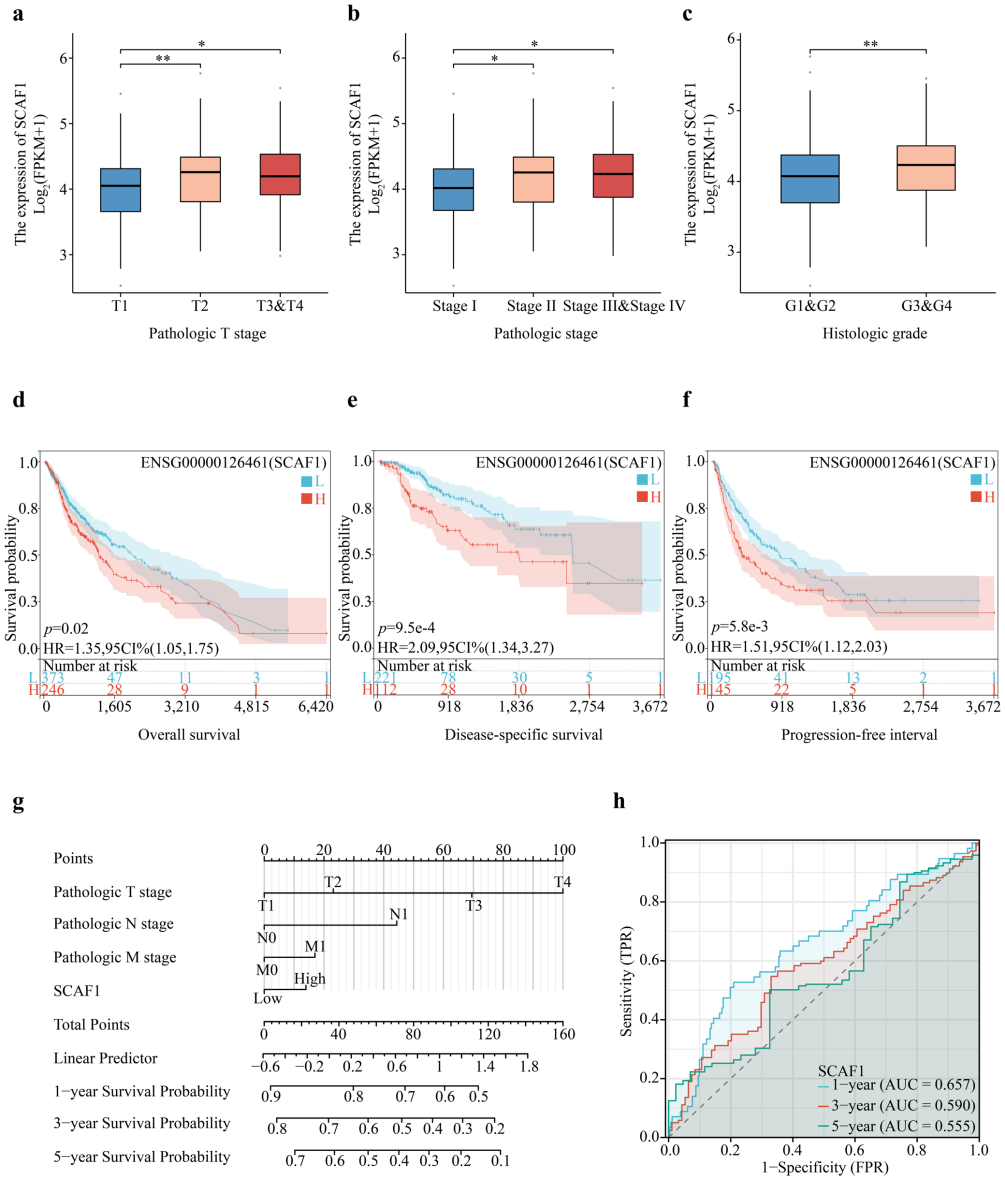
SCAF1's relationship with the prognosis of patients with HCC. (a–c) Increased expression levels of SCAF1 were notably associated with pathologic T stage, pathologic stage, and higher histologic grade. (d–f) HCC patients exhibiting reduced expression levels of SCAF1 demonstrated positive OS (HR = 1.35, *p* = 0.02), DSS (HR = 2.09, *p* = 9.5e‐4), and PFI (HR = 1.51, *p* = 5.8e‐3). (g) Nomograms and (h) ROC curves of SCAF1.

To investigate the relationship between clinical pathological characteristics and SCAF1, we observed changes in SCAF1 expression in HCC samples with different clinical pathological features. The results showed that as clinical staging, pathological staging, and histological staging increased, the overall expression of SCAF1 also increased (Figure 3a–c), indicating a close relationship between the clinical pathological characteristics of HCC patients and SCAF1 expression. We also examined the effect of SCAF1 expression on patient prognosis and utilised the Kaplan–Meier survival technique to estimate the OS, DSS, and PFI of HCC patients. The findings demonstrated that, in comparison to the low‐expression group, the high‐expression group of SCAF1 had reduced survival rates at each time (Figure 3d–f), suggesting that high SCAF1 expression may be associated with a poor prognosis in HCC patients. Furthermore, we created a prediction nomogram in which the T stage was the most significant contributor to OS to reliably forecast the survival rate of HCC patients (Figure 3g). We created a prognosis ROC curve, where the AUC values for 1, 3, and 5 years were all larger than 0.5, to further assess the precision of gene prediction (Figure 3h), confirming the high specificity and sensitivity of SCAF1 in predicting HCC prognosis and suggesting that SCAF1 expression is a key factor affecting the prognosis of HCC patients.

### 3.4 Potential Functions of SCAF1 in HCC

**FIGURE 4:**
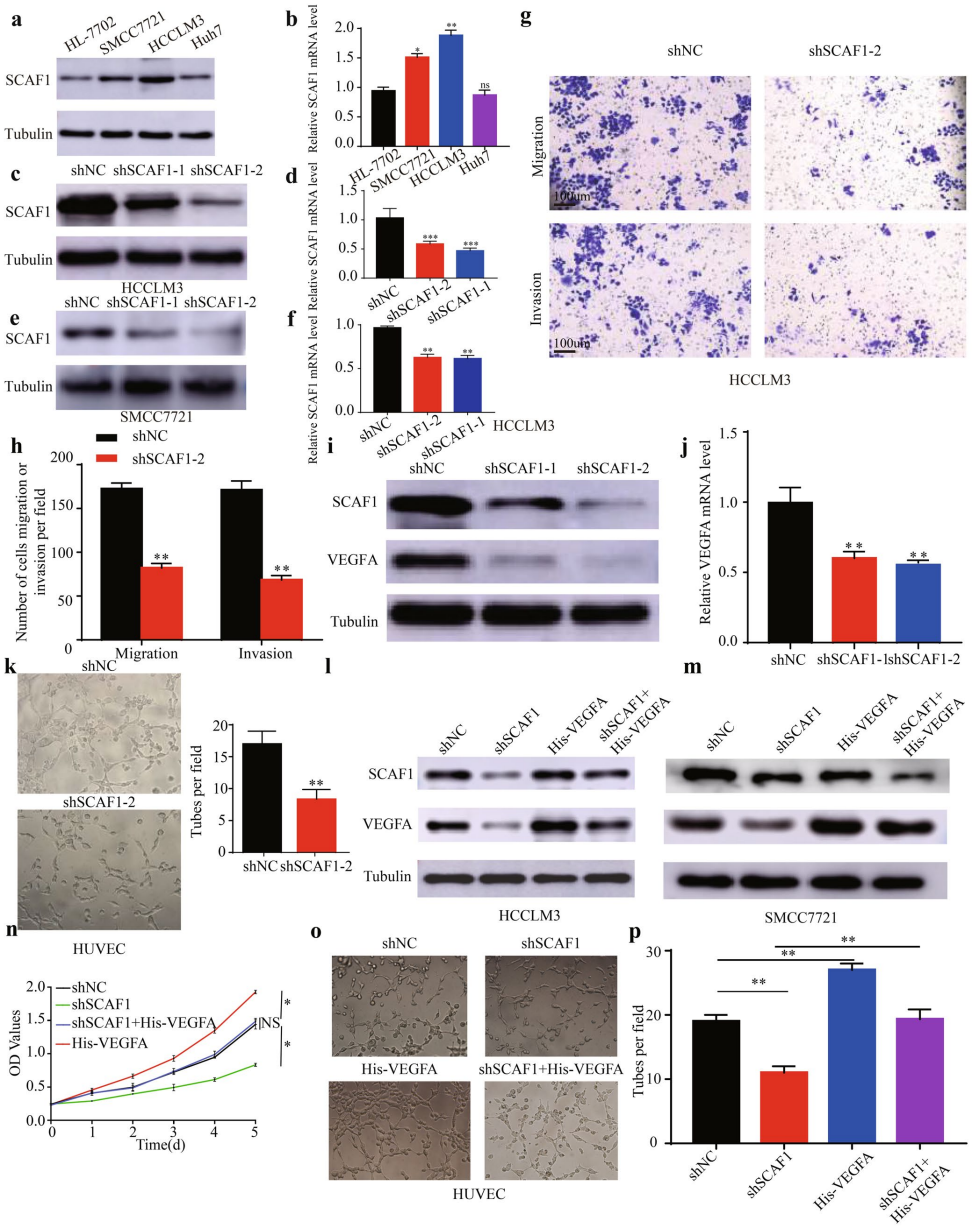
SCAF1 is associated with cell migration, invasion, and VEGF. (a) The WB experiment showed the protein expression of SCAF1 in different HCC cell lines. (b) Different HCC cell lines’ mRNA expression levels of SCAF1. (c, d) The protein and mRNA expression levels of SCAF1 under different inhibitors in HCCLM3. (e, f) The protein and mRNA expression levels of SCAF1 under different inhibitors in SMCC7721 (*p < 0.05, **p < 0.01, ***p < 0.001; NS, not significant). (g, h) The migration and invasion capabilities of HCCLM3 cells treated with either shSCAF1‐2 or shNC were individually assayed by Transwell. (i, j) The expression levels of SCAF1 and VEGFA under different inhibitors in HCCLM3 cells. (k) Tube‐formation assay in HUVECs treated with shSCAF1‐2. (l, m) The rescue test to ascertain the expression of SCAF1 and VEGFA in HCCLM3 and SMCC7721 cells. (n) CCK8 assays demonstrated that the upregulation of VEGFA significantly increased the proliferation of HCCLM3‐shSCAF1 cells. (o, p) Tube‐formation assay in HUVECs treated with shSCAF1, His‐VEGFA, or shSCAF1 + His‐VEGFA (*p < 0.05, **p < 0.01; NS, not significant).

Given the significant upregulation of SCAF1 expression levels in HCC, we aimed to study the regulatory mechanisms involving SCAF1 in HCC. According to (Figure S1f,g)'s GSEA analysis, the elevated DEGs linked to SCAF1 were primarily enriched in pathways pertaining to adhesion junctions and angiogenesis. This suggests that there may be a correlation between the expression of SCAF1 and the levels of metastasis and angiogenesis in HCC. To select suitable hepatocellular carcinoma cell lines for subsequent experiments, we conducted Western blot experiments on three hepatocellular carcinoma cell lines and one normal cell line. The hepatocellular carcinoma cell lines SMCC7721, HCCLM3, and Huh7 were compared to the normal liver tissue cell line HL‐7702 to determine the expression levels of SCAF1. We discovered that the expression level of SCAF1 was greatest in HCCLM3 (Figure 4a,b), indicating that this cell line is suitable for subsequent experiments. To explore the expression of SCAF1 in HCC, we obtained statistical information from the TCGA and ICGC websites. The results consistently demonstrated high expression of SCAF1 in HCC (Figure S1d,e). We indicate the close association of high SCAF1 expression with HCC, then we found both shSCAF1‐2 and shSCAF1‐1 were able to produce significant knockdown effects on SCAF1 expression levels in HCCLM3 as well as SMCC7702 cells (Figure 4c–f). In response to this conjecture, we carried out Transwell assays to evaluate the effect of SCAF1 knockdown on the invasive and migratory properties of HCC cells. We discovered that the SCAF1 knockdown group's HCCLM3 cells' capacity for migration and invasion was greatly diminished (Figure 4g,h). Previous research has demonstrated that angiogenesis plays a major role in the invasion and migration processes of tumour cells [28]. Combining our predicted results, we confirmed through WB experiments and mRNA detection that knocking down SCAF1 could reduce the degree of VEGFA expression in HCC (Figure 4i,j), and there was a notable decrease in the HUVECs' capacity to produce tubes in the angiogenesis experiment (Figure 4k). Through rescue experiments, we found that knockdown of SCAF1 was able to downregulate the expression level of VEGFA in both HCCLM3 and SMCC7721 cell lines compared to the control group. And overexpressing VEGFA, knockdown of SCAF1 expression was able to attenuate the effect of overexpression of VEGFA (Figure 4l,m). CCK8 assays further showed that down‐regulation of VEGFA significantly inhibited the growth of HCCLM3 cells, whereas overexpression of VEGFA reversed the growth inhibition induced by SCAF1 down‐regulation (Figure 4n). Besides, in tube formation assays, the angiogenic capacity was significantly reduced following SCAF1 down‐regulation, but this effect was alleviated by overexpressing VEGFA (Figure 4o,p). In conclusion, lowering SCAF1 may suppress the invasive migration of HCC cells by altering key signals (VEGFA) in the VEGF pathway.

### 3.5 Relationship Between SCAF1 and the Immune Microenvironment

**FIGURE 5:**
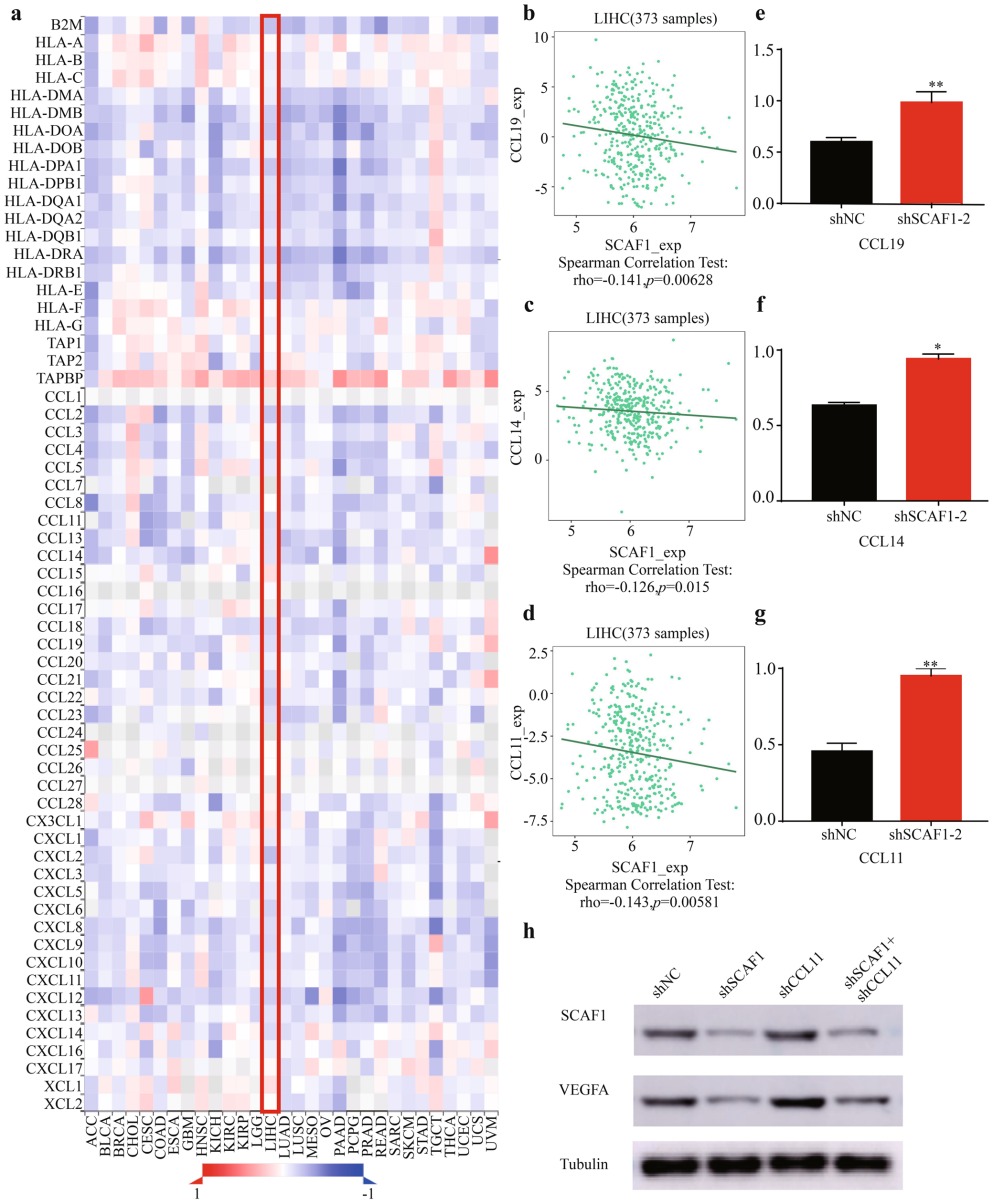
SCAF1 and cytokines. (a) The expression of different cytokines in cancers exhibiting high SCAF1 expression. (b–d) SCAF1 was linked to the CCL19, CCL14, and CCL11 expression by using TISIDB. (e–g) SCAF1 was negatively correlated with the expression of CCL19, CCL14, and CCL11. (h) The WB experiment demonstrated the relationship between SCAF1, CCL11, and VEGFA (*p < 0.05, **p < 0.01).

Chemokines and their receptors, a superfamily of secreted small molecules, are closely associated with the development of HCC [29]. Chemokines can regulate HCC cells by affecting the expression of VEGF [30]. To explore the relationship between the SCAF1 gene in HCC cells and chemokines, we used the TISIDB website to generate heatmaps for analysis, revealing that SCAF1 in HCC cells was associated with multiple chemokines, including CCL19, CCL14, and CCL11, and there was a negative correlation found between the expression levels of SCAF1 and the three chemokines (Figure 5a–d). Knocking down the expression of SCAF1 could increase the expression levels of CCL19, CCL14, and CCL11(Figure 5e–g). This implies that SCAF1 may influence HCC by controlling the expression of these three chemokines, which in turn influences the production of VEGF. Western blot analysis showed that SCAF1 silencing decreased VEGFA expression, whereas CCL11 knockdown increased VEGFA levels, but had no influence on the expression of SCAF1. Importantly, CCL11 knockdown rescued the suppressive effect of SCAF1 silencing on VEGFA expression (Figure 5h).

**FIGURE 6:**
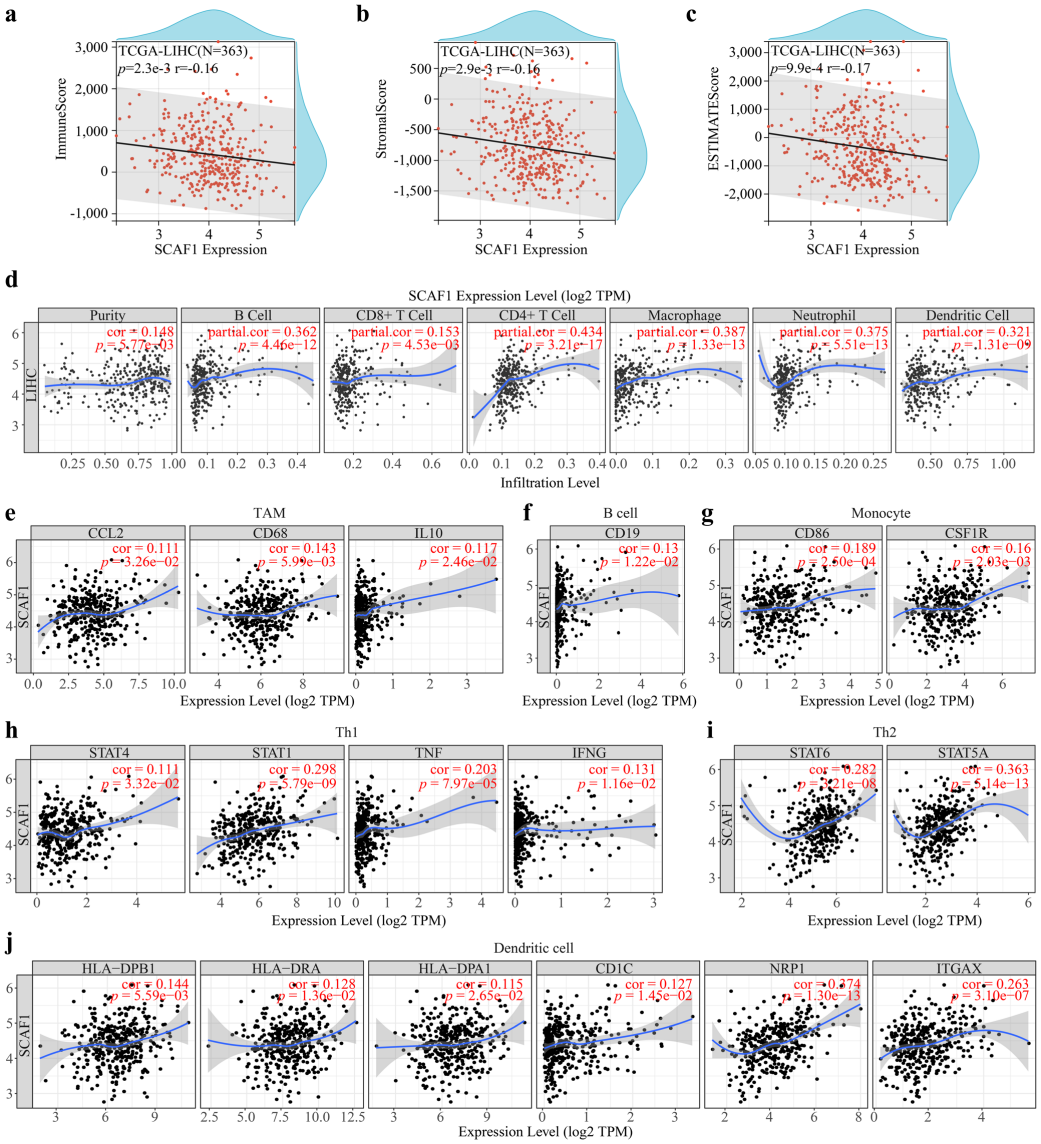
SCAF1 and immune infiltration. (a–c) Correlations between SCAF1 and immune score, stromal score and ESTIMATE score. (d) Correlation between SCAF1's expression and immune cells’ infiltration in HCC. SCAF1 expression is associated with gene markers of immune cells in HCC. (e) TAM; (f) B cell; (g) Monocyte; (h) Th1; (i) Th2; and (j) Dendritic cell.

Malignant tumour genesis, growth, and metastasis are all dependent on the tumour microenvironment (TME), which is made up of elements including VEGF and immune cells that have infiltrated the tumour [31, 32]. To further explore SCAF1's role in the tumour immune microenvironment and its relation with VEGF, we scored SCAF1 for immunity, stroma, and estimated and found that all three scores were negatively correlated with SCAF1 expression levels (Figure 6a–c). TIMER analysis indicated that higher SCAF1 expression was associated with increased infiltration of multiple immune cell types (including B cells, CD8+ and CD4+ T cells, neutrophils, macrophages, and dendritic cells) (Figure 6d). SCAF1 expression also positively correlated with markers of TAMs, B cells, monocytes, Th1/Th2 and DCs (Figure 6e–j), (Figure S2a–o), (Table S3 and Table S4). Combination therapy involving VEGF blockade therapy and immune checkpoint‐targeted immunotherapy is becoming increasingly important and has become a first‐line choice for various cancer treatments [33].

We observed a negative correlation between SCAF1 and several immune checkpoints (including CTLA‐4 and PD‐1) (Figure S1h), and high SCAF1 expression predicted poorer suitability for immunotherapy (Figure S1i,j). Comparing responders and non‐responders, enrichment differences were seen for B cells, MDSCs and CAFs (Figure S1h–m). Besides, SCAF1 is mainly expressed in the endothelial cells. Spatial transcriptomic analysis and cell clustering of tissue cells, combined with pseudotime trajectory analysis, recapitulated the characteristics of tumour heterogeneity and revealed the dynamic patterns of SCAF1 expression and cell type distribution (Figure S3d), (Figure S4c,d), (Figure S4e). Post‐immunotherapy, VEGFA and SCAF1 were suppressed in responders (Figure S4a,b), while CD274 increased and CAF/MDSC levels decreased in the responsive group (Figure S1i–k), indicating immunotherapy markedly reshapes immune composition in HCC.

### 3.6 Small Molecule Drugs Targeting SCAF1

**FIGURE 7:**
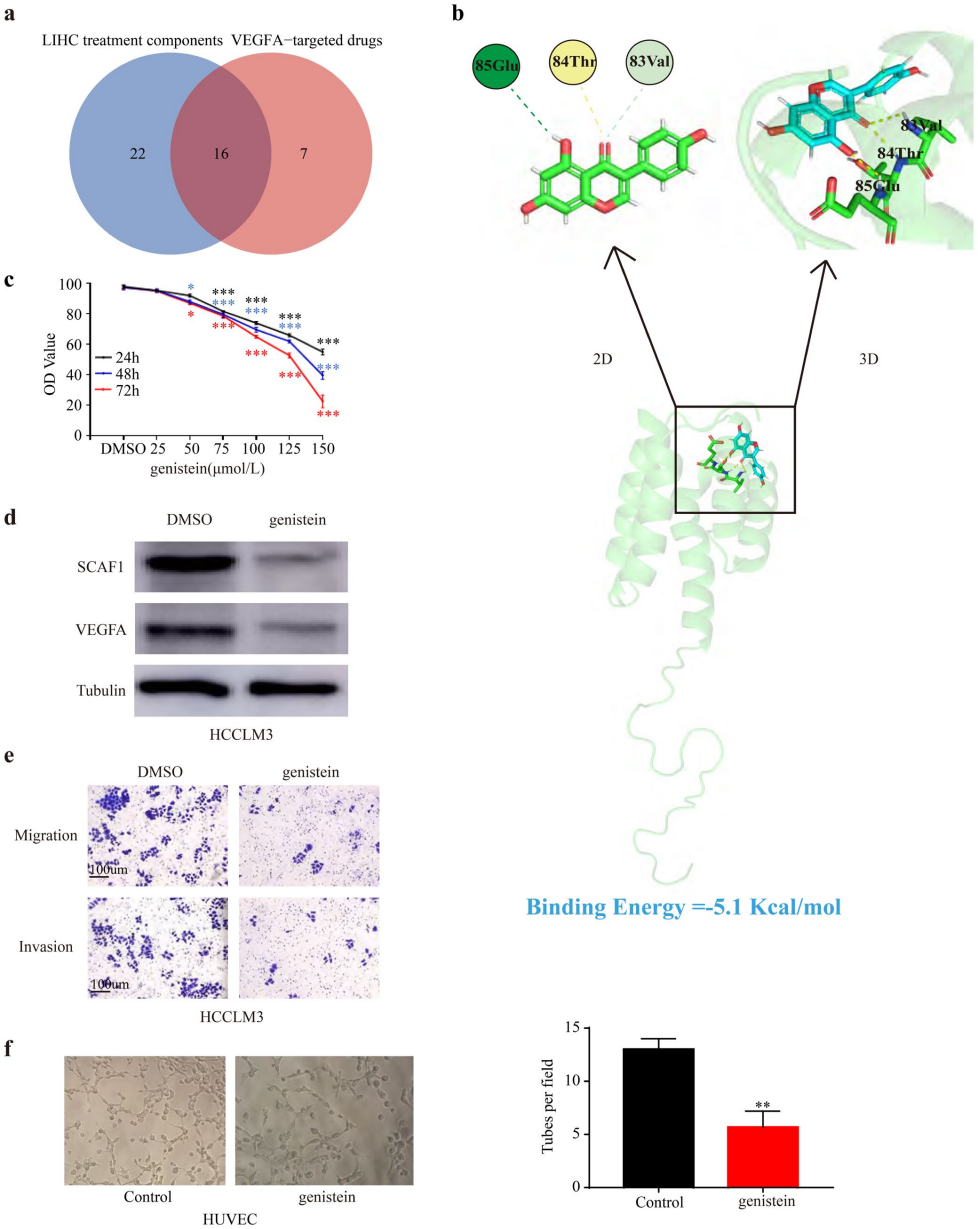
Screening of micromolecular drugs acting on SCAF1. (a) The therapeutic molecules of HCC and the drugs targeting VEGFA were intersected to obtain 16 kinds of small molecules, as seen in the Venn diagram. (b) Mechanism of interaction between genistein and SCAF1. (c) Effects of different times and concentrations of genistein on cell proliferation. (d) The WB experiment demonstrated the impact of genistein on SCAF1 and VEGFA expression. (e) Genistein affects the migration and invasion ability of HCCLM3 cells. (f) Tube‐formation assay in HUVECs treated with genistein or not (*p < 0.05, **p < 0.01, ***p < 0.001).

Through network pharmacology analysis, we explored small‐molecule drugs associated with the treatment of hepatocellular carcinoma (HCC) and drugs targeting VEGFA. By searching the TCMSP database and cross‐analysing HCC therapeutic molecules with drugs targeting VEGFA, we identified 16 small molecules (Figure 7a). Using molecular docking techniques, we modelled the interactions between these small molecules and the SCAF1 protein (Figure 7b). Genistein exhibited the strongest binding energy (−5.1 Kcal/mol) with SCAF1 (Figure S5a–d), (Table S2), interacting with three amino acid residues of SCAF1 (85Glu, 84Thr, and 83Val) through hydrogen bonding, demonstrating stronger binding affinity compared to other molecules. Compared with the DMSO group, cell viability decreased when the concentration of genistein and the time of administration increased. We chose the concentration of genistein for the treatment of HCCLM3 cells to be 100 μmol/L to ensure cell viability of around 70% (Figure 7c). Western blot experiments demonstrated that genistein significantly reduced the expression levels of SCAF1 and VEGFA (Figure 7d). Furthermore, the migration and invasion capabilities of HCC cells were markedly reduced upon genistein treatment (Figure 7e). Additionally, Genistein treatment significantly reduced the angiogenic density of HUVECs compared to the control group (Figure 7f). These findings suggest that genistein inhibits the occurrence and development of HCC by suppressing the expression levels of SCAF1, thereby downregulating key molecules in the angiogenesis process.

## 4 Discussion

Hepatocellular carcinoma (HCC) is a common cancer and ranks second in terms of mortality globally. While the incidence of HCC has been somewhat controlled recently and remains relatively stable, the prognosis for patients still remains concerning. Due to HCC's typical highly vascularised and arterialised solid tumour nature, anti‐angiogenic therapies have been considered effective strategies in its treatment. Drugs targeting VEGF, e.g., sorafenib, have been widely utilised in clinical practice for HCC patients [34]. However, in diagnostic practice, due to the lack of biomarkers predicting VEGF, both OS and PFS for patients have not been effectively improved. Consequently, there is an urgent need to establish new biomarkers related to angiogenesis in HCC [35, 36].

In our study, we used WGCNA to identify genes linked to HCC progression and focused on modules related to angiogenesis, focal adhesion, and EMT. Using 101 algorithmic combinations from 10 machine‐learning methods, we built a prognostic risk model that reached a C‐index of 0.763 across three datasets. Single‐cell RNA‐seq from GEO revealed four major cell types (T cells, endothelial cells, macrophages, hepatocytes); intersecting their DEGs with model genes yielded eight overlaps, including the seldom‐reported SCAF1. Subsequent expression, clinicopathological, survival, and functional analyses showed SCAF1 is overexpressed in HCC, associates with advanced stage and poor prognosis, and likely promotes angiogenesis and tumour invasion.

It is known that VEGF can heighten the invasion and migration ability of HCC through various regulatory axes [37, 38, 39]. Our results suggest that SCAF1 not only promotes angiogenesis in HCC but also enhances the migration and invasion levels of HCC. We speculate that SCAF1 may increase cancer cell metastasis through the VEGF pathway.

In previous studies, the production status of chemokines in the TME provided a predictive means for VEGF levels in cancer [13, 40]. For example, CCL19 inhibits angiogenesis in colorectal cancer by inhibiting the Met/ERK/Elk‐1/HIF‐1α/VEGF‐A pathway [17, 41]. Our experimental results indicate that knocking down SCAF1 can promote the expression of CCL19, CCL14, and CCL11. We propose that elevated SCAF1 expression may promote neovascularisation in HCC by downregulating anti‐angiogenic cytokines.

Additionally, the level of immune infiltration in the TME can affect the angiogenesis level in HCC. Immunosuppressive cells such as regulatory T cells and TAMs can exert immunosuppressive activity, regulate downstream signalling pathways, and drive angiogenesis [42]. SCAF1 may be involved in multiple tumour immune mechanisms [43], such as inducing the expression of SCAF1 in GBMLGG, which can promote the M2 polarisation of macrophages, thereby shifting macrophage subtypes unfavourably for patient survival. In our study, we used immune scores, stromal scores, and ESTIMATE scores to quantify in situ cell infiltration [44]. The results point out that high expression of SCAF1 reduces the immune scores of HCC and contributes to the recruitment of CD8+ T cells, macrophages, and other immune cells. Using the TIMER database, we analysed the expression of SCAF1 and immune cell markers in HCC. As expected, the expression of SCAF1 is positively associated with various immune regulatory cells (TAMs, B cells, Th1, Th2, dendritic cells), indicating that SCAF1 may drive the angiogenesis pathway by inducing the anti‐immune activity of these cells.

Small molecule‐related heterologous therapies have been preliminarily applied in the treatment of HCC patients [45, 46, 47], and patients treated with traditional Chinese medicine as adjunctive therapy have shown relatively significant improvements in prognosis. Genistein is a common precursor for the synthesis of antimicrobial and antitumor phytoalexins in plants of the Leguminosae family. It has a range of potential health benefits, including chemoprevention of HCC, breast cancer, and prostate cancer [48, 49, 50]. Guo et al. showed that genistein exerts anti‐tumour effects in prostate cancer by inhibiting angiogenesis [51], but reports on the anti‐tumour mechanism of genistein in HCC are incomplete. In our study, we found that genistein can form a stable docking with the SCAF1 molecule and inhibit the proliferation of HCCLM3 cells. Furthermore, genistein can inhibit the expression of SCAF1 and VEGFA, as well as the invasion and migration capabilities of HCC cells. And the angiogenic capacity of cells treated with genistein was significantly reduced. We speculate that genistein may inhibit angiogenesis in HCC by binding to SCAF1 and suppressing its expression, thereby suppressing the occurrence and development of HCC.

Although we performed meta‐analyses across TCGA, ICGC, and GEO and obtained promising therapeutic clues, the clinical efficacy of the identified small molecules still requires validation, and the VEGF‐blocking effect predicted from SCAF1 should be confirmed in patient cohorts. While we characterised the temporal and spatial distribution of SCAF1 in liver tissue, additional in vivo studies are needed to validate these findings and optimise treatment strategies.

## 5 Conclusion

In summary, we identified a biomarker, SCAF1, that can affect the angiogenic status of HCC patients and found that SCAF1 can act as an oncogene, leading to poor prognosis of HCC patients. High expression of SCAF1 in HCC can lead to a poor immune microenvironment as well as up‐regulation of angiogenic‐related cytokines' expression. Meanwhile, high expression of SCAF1 suggests poor immunotherapy and is closely associated with genistein, an active ingredient capable of anti‐tumour angiogenesis. All these results suggested that SCAF1 can be used as a diagnostic and therapeutic biomarker for HCC patients and provide a reference for updating the therapeutic regimen of HCC patients.

## Author Contributions

**Xiaoyu Qu:** writing – original draft, writing – review and editing, visualization, data curation. **Hao Wan:** data curation, visualization, writing – review and editing, writing – original draft. **Ruiyu Zhang:** data curation, writing – original draft, writing – review and editing, visualization. **Chenshuo Fang:** writing – original draft, visualization, writing – review and editing, data curation. **Hao Zheng:** conceptualization, methodology, software, validation, formal analysis, data curation, writing – original draft, writing – review and editing, visualization, supervision, project administration. **Shengwei Tang:** conceptualization, writing – original draft, methodology, validation, visualization, writing – review and editing, software, formal analysis, project administration, data curation, supervision. **Xin Wang:** writing – original draft, visualization, writing – review and editing, data curation. **Zichuan Yu:** conceptualization, writing – original draft, writing – review and editing, visualization, validation, methodology, software, formal analysis, project administration, supervision, data curation. **Da Huang:** conceptualization, project administration. **Gen Chen:** conceptualization, project administration. **Ye Zhu:** writing – original draft, writing – review and editing, visualization, data curation. **Ruida Shi:** conceptualization, writing – original draft, methodology, validation, visualization, writing – review and editing, software, formal analysis, project administration, data curation, supervision.

## Funding

This study was supported by the Science and Technology Foundation of Guizhou Provincial Health Commission (gzwkj2023‐039 and YKLPGEX202401) and Doctoral Foundation of Guizhou Provincial People’s Hospital (GZSYBS[2022]05).

## Ethics Statement

This study was approved by the Ethics Committee of the Second Affiliated Hospital of Nanchang University (ID: lT‐2024‐294).

## Conflicts of Interest

The authors declare no conflicts of interest.

## Supporting information


**Figure S1:** (a) Determining the optimal soft threshold. (b) Identification of HCC‐related modules by WGCNA. (c) Gene correlation scatter plot of the turquoise module. (d, e) Analysis of SCAF1 expression in tumour and normal tissues utilising TCGA and ICGC databases. GSEA was employed to confirm the gene signatures, involving positive regulation of (f) VEGF signalling pathway and (g) focal adhesion. (h) Correlation between SCAF1 and various immune checkpoints in HCC. (i) Relationship between SCAF1 and TMB. (j) High SCAF1 expression was associated with high TIDE scores. (k–m) Expression of CAF, MDSC and CD274 in responders and non‐responders to immunotherapy (**p < 0.01, ***p < 0.001, ****p < 0.0001; NS, not significant).

**Figure S2:** Relationship of SCAF1 expression with gene markers of immune cells in HCC. (a) M1; (b) Natural killer cell; (c) Neutrophils; (d) Treg; (e) Tfh; (f) Th17; (g) Resting Treg; (h) Effector Treg T‐cell; (i) Naïve T‐cell; (j) Effector T‐cell; (k) Exhausted T‐cell; (l) Th1‐like; (m) Memory T‐cell; (n) Effector memory T‐cell; and (o) Resident memory T‐cell.

**Figure S3:** Spatial transcriptomics and single‐cell sequencing. (a) Single‐cell dimensionality reduction distribution of 34 liver cancer patients. (b) Visualisation of the feat15 gene. (c) The dotplot shows the expression differentiation of SCAF1 in the 4 types of cells. (d) Liver tissue sections and cell reclustering by the UMAP method.

**Figure S4:** Further study of the post‐immunotherapy tumour microenvironment in patients with HCC. (a) SCAF1 and VEGFA expression in HCC cell lines. (b) SCAF1 and VEGFA's expression in HCC cell lines after immunotherapy. (c) tSNE cell cluster analysis was carried out on the cells after treatment. (d) Further clustering of one of the cell types into six clusters. (e) Changes in SCAF1 expression levels and cell types using pseudotime.

**Figure S5:** Molecular docking analysis of SCAF1 and the main ingredients of traditional Chinese medicines. (a) Beta‐carotene, (b) Progesterone, (c) Baicalein, and (d) Luteolin.

**Table S1:** Primers target sequences.

**Table S2:** Molecular docking binding energies (kJ/mol) of SCAF1.

**Table S3:** Correlation analysis between SCAF1 and gene markers of different types of immune cells in TIMER.

**Table S4:** Correlation analysis between SCAF1 and gene markers of different types of T cells in TIMER.

**Table S5:** jcmm71203‐sup‐0010‐TableS5.xlsx.

## Data Availability

The data that support the findings of this study are available from the corresponding author upon reasonable request.
