# Supplementary figures and images for: Deciphering Angiogenic Drivers in Hepatocellular Carcinoma: From Prognostic Signature Construction to Genistein‐Mediated Inhibition
Source: J Cell Mol Med. 2026 May 24;30(10):e71203. doi: 10.1111/jcmm.71203 (PMC13238579; doi:10.1111/jcmm.71203)

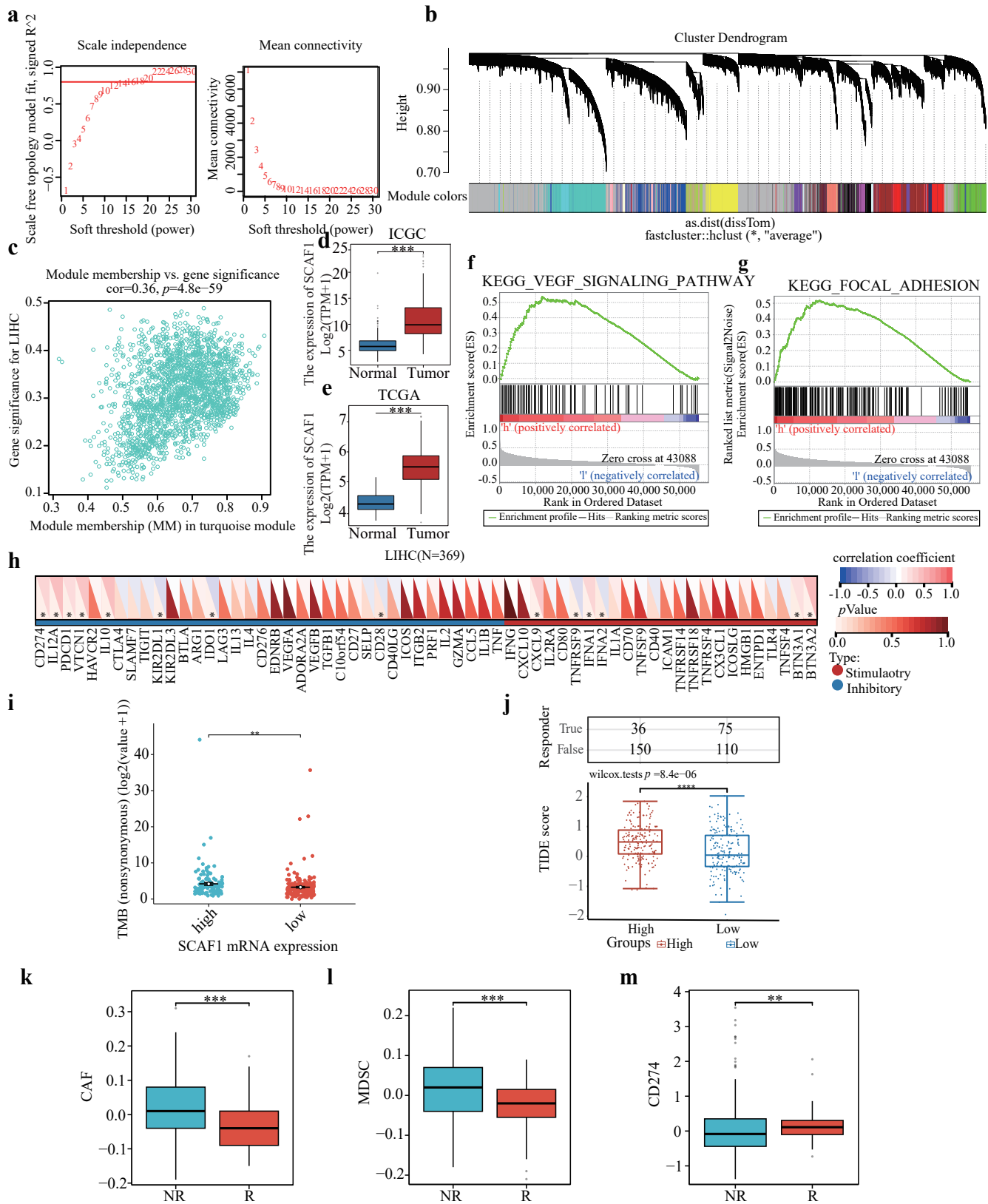

Supplement: Supplementary file 1 — Figure S1: (a) Determining the optimal soft threshold. (b) Identification of HCC‐related modules by WGCNA. (c) Gene correlation scatter plot of the turquoise module. (d, e) Analysis of SCAF1 expression in tumour and normal tissues utilising TCGA and ICGC databases. GSEA was employed to confirm the gene signatures, involving positive regulation of (f) VEGF signalling pathway and (g) focal adhesion. (h) Correlation between SCAF1 and various immune checkpoints in HCC. (i) Relationship between SCAF1 and TMB. (j) High SCAF1 expression was associated with high TIDE scores. (k–m) Expression of CAF, MDSC and CD274 in responders and non‐responders to immunotherapy (**p < 0.01, ***p < 0.001, ****p < 0.0001; NS, not significant). [file JCMM-30-e71203-s007.pdf]

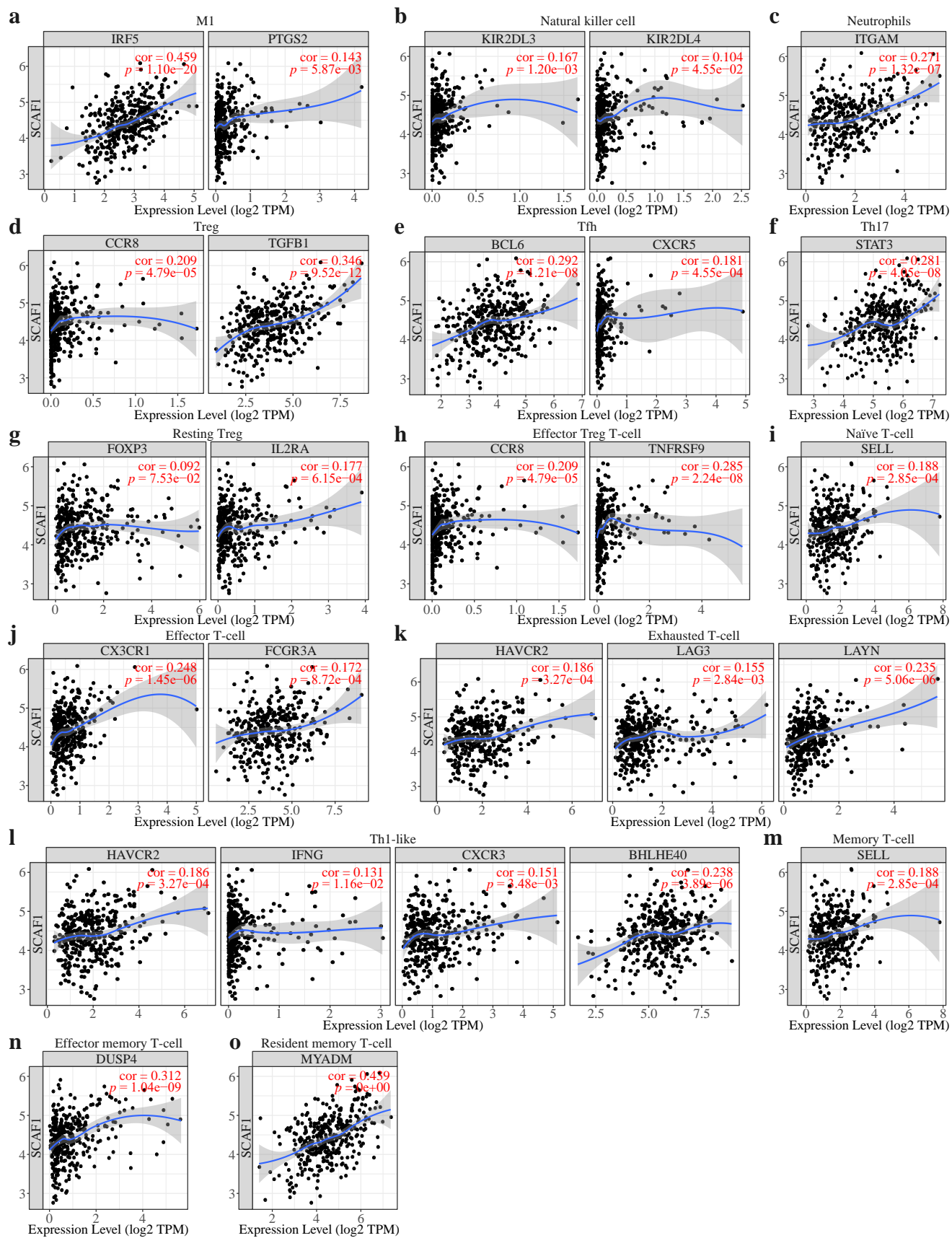

Supplement: Supplementary file 2 — Figure S2: Relationship of SCAF1 expression with gene markers of immune cells in HCC. (a) M1; (b) Natural killer cell; (c) Neutrophils; (d) Treg; (e) Tfh; (f) Th17; (g) Resting Treg; (h) Effector Treg T‐cell; (i) Naïve T‐cell; (j) Effector T‐cell; (k) Exhausted T‐cell; (l) Th1‐like; (m) Memory T‐cell; (n) Effector memory T‐cell; and (o) Resident memory T‐cell. [file JCMM-30-e71203-s001.pdf]

**a**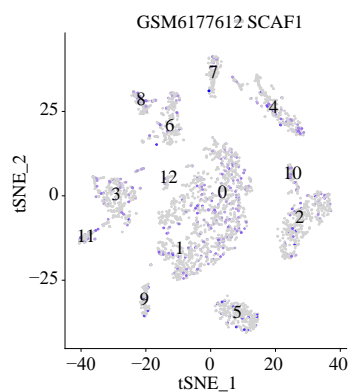

GSM6177612 VEGFA

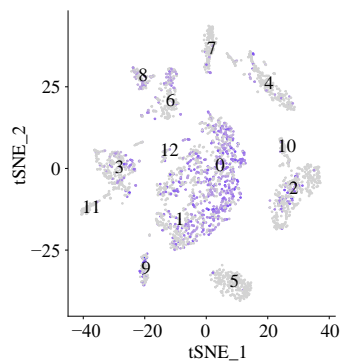**b**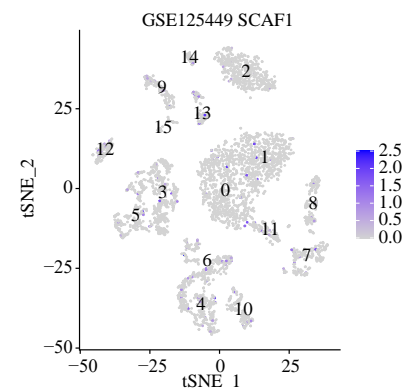

GSE125449 VEGFA

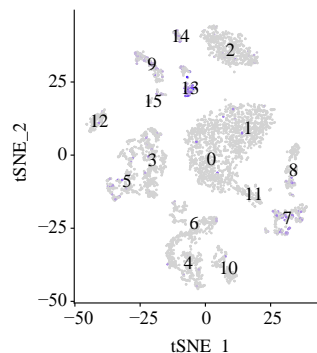**c**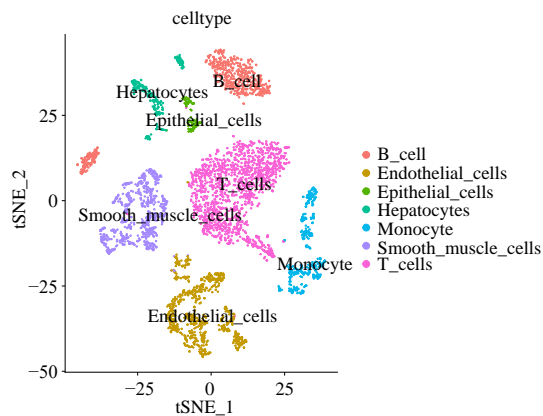**d**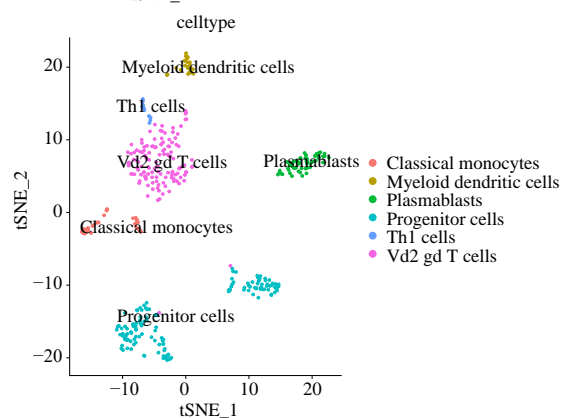**e**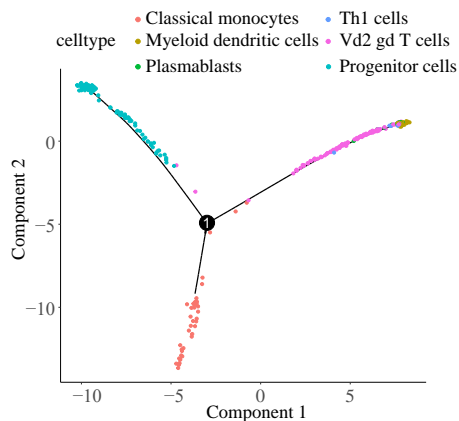

SCAF1

0.00 0.25 0.50 0.75 1.00

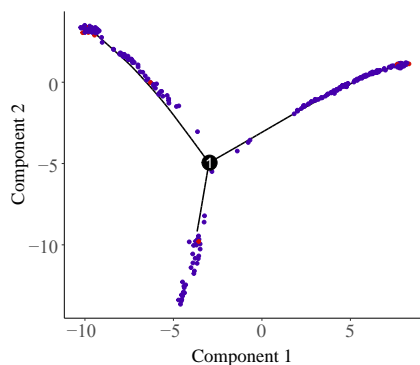

Pseudotime

0 5 10 15 20

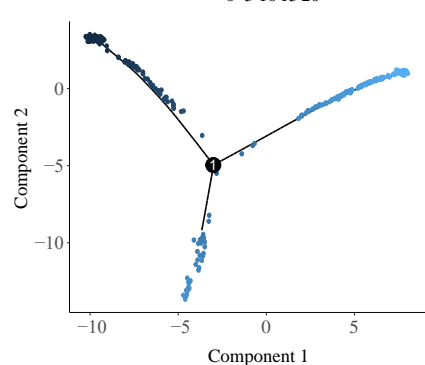

Supplement: Supplementary file 4 — Figure S4: Further study of the post‐immunotherapy tumour microenvironment in patients with HCC. (a) SCAF1 and VEGFA expression in HCC cell lines. (b) SCAF1 and VEGFA's expression in HCC cell lines after immunotherapy. (c) tSNE cell cluster analysis was carried out on the cells after treatment. (d) Further clustering of one of the cell types into six clusters. (e) Changes in SCAF1 expression levels and cell types using pseudotime. [file JCMM-30-e71203-s002.pdf]

**a**

beta-carotene

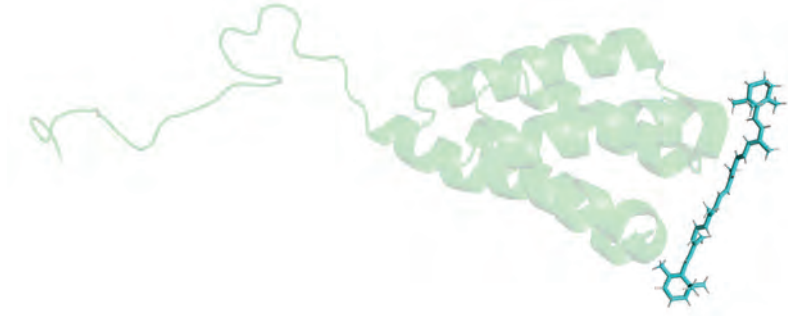

**b**

progesterone

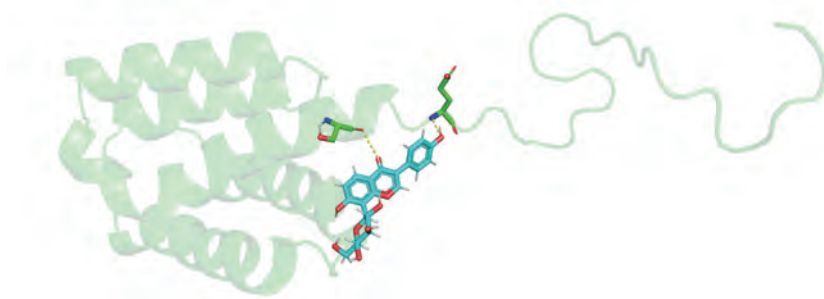

**c**

baicalein

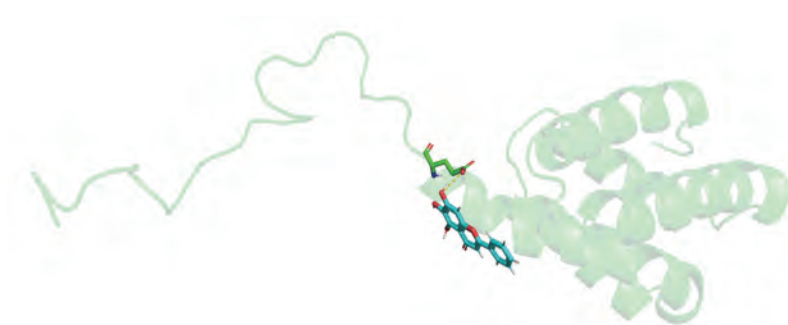

**d**

luteolin

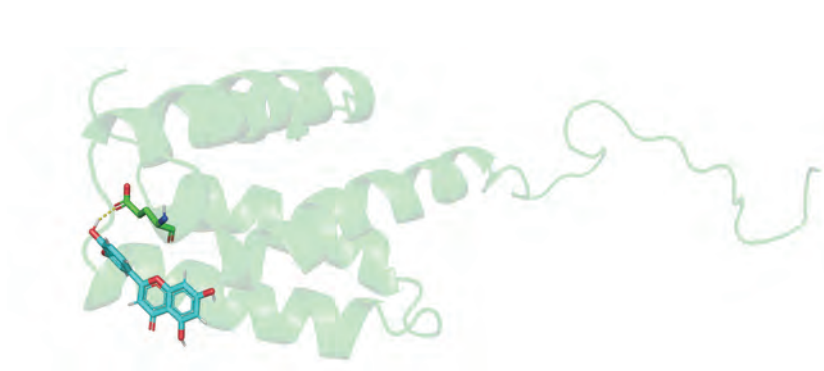

Supplement: Supplementary file 5 — Figure S5: Molecular docking analysis of SCAF1 and the main ingredients of traditional Chinese medicines. (a) Beta‐carotene, (b) Progesterone, (c) Baicalein, and (d) Luteolin. [file JCMM-30-e71203-s010.pdf]
