# Supplementary material for: Deciphering Angiogenic Drivers in Hepatocellular Carcinoma: From Prognostic Signature Construction to Genistein‐Mediated Inhibition
Source: J Cell Mol Med. 2026 May 24;30(10):e71203. doi: 10.1111/jcmm.71203 (PMC13238579; doi:10.1111/jcmm.71203)
Supplement: Supplementary file 3 — Figure S3: Spatial transcriptomics and single‐cell sequencing. (a) Single‐cell dimensionality reduction distribution of 34 liver cancer patients. (b) Visualisation of the feat15 gene. (c) The dotplot shows the expression differentiation of SCAF1 in the 4 types of cells. (d) Liver tissue sections and cell reclustering by the UMAP method. [file JCMM-30-e71203-s003.pdf]

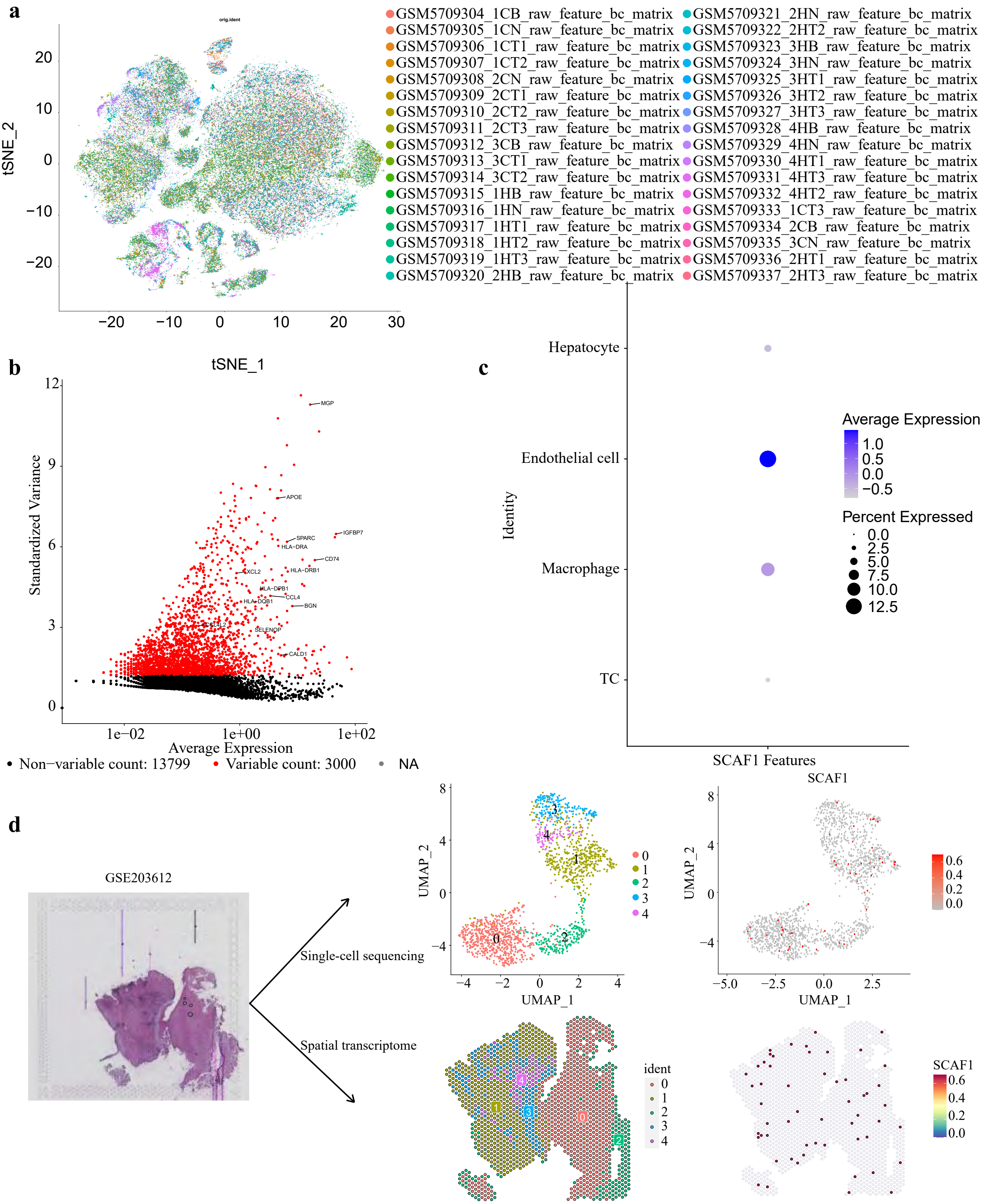
a
orig.ident
tSNE_2
tSNE_1
GSM5709304_1CB_raw_feature_bc_matrix
GSM5709305_1CN_raw_feature_bc_matrix
GSM5709306_1CT1_raw_feature_bc_matrix
GSM5709307_1CT2_raw_feature_bc_matrix
GSM5709308_2CN_raw_feature_bc_matrix
GSM5709309_2CT1_raw_feature_bc_matrix
GSM5709310_2CT2_raw_feature_bc_matrix
GSM5709311_2CT3_raw_feature_bc_matrix
GSM5709312_3CB_raw_feature_bc_matrix
GSM5709313_3CT1_raw_feature_bc_matrix
GSM5709314_3CT2_raw_feature_bc_matrix
GSM5709315_1HB_raw_feature_bc_matrix
GSM5709316_1HN_raw_feature_bc_matrix
GSM5709317_1HT1_raw_feature_bc_matrix
GSM5709318_1HT2_raw_feature_bc_matrix
GSM5709319_1HT3_raw_feature_bc_matrix
GSM5709320_2HB_raw_feature_bc_matrix
GSM5709321_2HN_raw_feature_bc_matrix
GSM5709322_2HT2_raw_feature_bc_matrix
GSM5709323_3HB_raw_feature_bc_matrix
GSM5709324_3HN_raw_feature_bc_matrix
GSM5709325_3HT1_raw_feature_bc_matrix
GSM5709326_3HT2_raw_feature_bc_matrix
GSM5709327_3HT3_raw_feature_bc_matrix
GSM5709328_4HB_raw_feature_bc_matrix
GSM5709329_4HN_raw_feature_bc_matrix
GSM5709330_4HT1_raw_feature_bc_matrix
GSM5709331_4HT3_raw_feature_bc_matrix
GSM5709332_4HT2_raw_feature_bc_matrix
GSM5709333_1CT3_raw_feature_bc_matrix
GSM5709334_2CB_raw_feature_bc_matrix
GSM5709335_3CN_raw_feature_bc_matrix
GSM5709336_2HT1_raw_feature_bc_matrix
GSM5709337_2HT3_raw_feature_bc_matrix
b
Standardized Variance
Average Expression
MGP
APOE
IGFBP7
SPARC
HLA-DRA
CD74
CXCL2
HLA-DRB1
HLA-DPB1
CCL4
HLA-DQB1
BGN
SELENOP
CALD1
Non−variable count: 13799
Variable count: 3000
NA
c
Identity
Hepatocyte
Endothelial cell
Macrophage
TC
SCAF1 Features
Average Expression
Percent Expressed
d
GSE203612
Single-cell sequencing
Spatial transcriptome
UMAP_1
UMAP_2
SCAF1
ident
