# Supplementary material for: Deciphering Angiogenic Drivers in Hepatocellular Carcinoma: From Prognostic Signature Construction to Genistein‐Mediated Inhibition
Source: J Cell Mol Med. 2026 May 24;30(10):e71203. doi: 10.1111/jcmm.71203 (PMC13238579; doi:10.1111/jcmm.71203)
Supplement: Supplementary file 6 — Table S1: Primers target sequences. [file JCMM-30-e71203-s006.doc]

**Supplementary Table.1** Primers target sequences

| name | sequence | accession number |
| --- | --- | --- |
| Primers for real-time PCR: |  |  |
| SCAF1 forward | 5'- GAGCCGCAGCCCGAG -3′ | NM_021228.3 |
| SCAF1 reverse | 5'- TGGGATCTCTGTCCCCGATT-3′ |  |
| VEGFA forward | 5'-TGCGGATCAAACCTCACCAA-3′ | NM_001171623.2 |
| VEGFA reverse | 5'-GGCTCCAGGGCATTAGACAG-3′ |  |
| CCL19 forward | 5’- GAAGACTGCTGCCTGTCTGT-3’ | NM_006274.3 |
| CCL19 reverse | 5’-GCAGTCTCTGGATGATGCGT -3’ |  |
| CCL11 forward | 5’- GTGGGTGCAGGATTCCATGA-3’ | NM_002986.3 |
| CCL11 reverse | 5’-CCCATGCCCTTTGGACTGAT -3’ |  |
| CCL14 forward | 5’- GGGGGAAACCGAAGGTTGTT-3’ | NM_032962.5 |
| CCL14 reverse | 5’- AGACAATTCCGGGCTTGGAG -3’ |  |
